# Identification of Novel *Neisseria gonorrhoeae* Lineages Harboring Resistance Plasmids in Coastal Kenya

**DOI:** 10.1093/infdis/jiy240

**Published:** 2018-04-26

**Authors:** Ana Cehovin, Odile B Harrison, Steven B Lewis, Philip N Ward, Caroline Ngetsa, Susan M Graham, Eduard J Sanders, Martin C J Maiden, Christoph M Tang

**Affiliations:** 1 Sir William Dunn School of Pathology, University of Oxford, United Kingdom; 2 Department of Zoology, University of Oxford, United Kingdom; 3 Nuffield Department of Medicine, University of Oxford, United Kingdom; 4 Kenya Medical Research Institute–Wellcome Trust Research Programme, Kilifi, Kenya; 5 University of Washington, Seattle; 6 Department of Global Health, University of Amsterdam, The Netherlands

**Keywords:** *Neisseria gonorrhoeae*, antimicrobial resistance, whole-genome sequencing, MSM, plasmids

## Abstract

**Background:**

Africa has the highest incidence of gonorrhea in the world. However, little is known about gonococcal populations in this continent or mechanisms of antimicrobial resistance (AMR).

**Methods:**

Whole-genome sequence data were analyzed from 103 *Neisseria gonorrhoeae* isolates from 73 patients, mainly men who have sex with men, from coastal Kenya. We annotated loci, defined the core genome, defined mechanisms of AMR, and performed phylogenetic analysis. For patients with multiple episodes of gonorrhea, we determined whether infections occurred with related strains.

**Results:**

We identified 3 clusters of isolates that are phylogenetically distinct from isolates found elsewhere. Plasmids were virtually ubiquitous: pTetM and p*bla*TEM were found in 97%, and 55% of isolates, respectively. This was associated with high doxycycline use for undiagnosed sexually transmitted infections. Twenty-three percent of multiple episodes of gonorrhea in the same individual were caused by a related strain, suggesting inadequate treatment or reinfection.

**Conclusions:**

The prevalence of plasmid-mediated AMR in Kenyan gonococci contrasts with that in wealthy countries, where AMR is largely chromosomally mediated. Antimicrobials have a profound effect on the maintenance of lineages harboring plasmids. Doxycycline can select for tetracycline and penicillin resistance, through plasmid cooperation. Understanding the mechanisms of AMR in high-risk groups is required to inform treatment strategies.

## Teaser

Lineages of *Neisseria gonorrhoeae* in coastal Kenya are phylogenetically distinct from those elsewhere in the world, and a high proportion possess plasmids. Antimicrobial use can shape gonococcal population structure and maintain lineages that carry and transmit resistance plasmids.

Sexually transmitted infection (STI) caused by *Neisseria gonorrhoeae* is a major public health concern [1]. Complications from gonococcal disease include infertility, pelvic inflammatory disease, ectopic pregnancy and neonatal conjunctivitis, which can cause blindness [2]. Furthermore, gonococcal infection increases HIV acquisition and transmission by increasing viral shedding [3]. Effective treatment of *N. gonorrhoeae* infection is therefore essential. However, the bacterium has developed resistance against all available classes of antimicrobials [2] and recently was added to the World Health Organization (WHO) list of highly antibiotic-resistant, priority pathogens [4]. While most AMR determinants exist on the chromosome, penicillin and tetracycline resistance can be conferred by plasmids (p*bla*TEM and pTetM), which can rapidly spread through gonococcal populations [5]; p*bla*TEM can be mobilized between gonococci by the conjugative plasmid pTetM [6]. Understanding the molecular epidemiology of gonococcal infection, including the development and acquisition of AMR, is therefore essential for effective management of gonococcal infection [2].

Africa has the highest incidence of gonorrhea in the world, with an estimated 50–60 new infections per 1000 adults per year, compared with an annual incidence of 7–8 infections per 1000 adults in Europe [7]. Despite this, remarkably little is known about the populations of *N. gonorrhoeae* that circulate in Africa. Furthermore, there is little information on the extent and mechanisms of gonococcal AMR in strains from Africa. In Kenya, there is a high prevalence of gonococcal infection among men who have sex with men (MSM) who are at high risk of acquiring STIs [8]. Core transmission groups such as sex workers and MSM in particular have been shown to have a critical role in the emergence and transmission of gonococcal AMR [9]. Therefore, monitoring and treatment of high-risk groups is essential to understand transmission of the bacterium and inform strategies for its control [9].

Recent advances in whole-genome sequencing (WGS) have provided insights into the global spread of successful gonococcal lineages and the molecular mechanisms responsible for AMR. These studies have focused on gonococcal disease in high-income countries [10, 11], where diagnostic facilities and therapeutic options are different from those in less wealthy countries in sub-Saharan Africa.

Here we characterized *N. gonorrhoeae* isolates predominantly from MSM in coastal Kenya by WGS. Results reveal that gonorrhea in this high-risk group is caused by a unique population of gonococcal lineages not found in other parts of the world. In contrast to Europe and the United States, where AMR is predominantly chromosomally mediated [10, 11], AMR in gonococci from a high-risk group in coastal Kenya is mainly conferred through the acquisition of plasmids p*bla*TEM and pTetM. This is associated with high doxycycline use for undiagnosed STIs. Our findings demonstrate how antibiotic policies influence gonococcal populations through selective pressure and have significant implications for the management of gonococcal disease and other STIs in resource-poor settings.

## MATERIALS AND METHODS

### Clinical Setting and Samples

Between June 2010 and May 2015, 103 *N. gonorrhoeae* isolates were obtained from 73 patients (aged 18–49 years), including sex workers and MSM, who were participating in cohort studies at the Kenyan Medical Research Institute clinic in Mtwapa. Ethical approval was granted by the Kenya Medical Research Institute (KEMRI) Scientific and Ethical Review Unit (approval 2842). Gonorrhea was diagnosed in men with urethral or rectal discharge, in men who reported receptive anal intercourse, and in women irrespective of symptoms. Samples were obtained by swabbing and screening for *N. gonorrhoeae* by Gram stain, the oxidase test, and API-NH (bioMerieux, France). A total of 31 of 73 patients (42.5%) were HIV positive. Of 103 *N. gonorrhoeae* isolates, 84 (81.6%) were urethral, 17 (16.5%) were rectal, and 2 (1.9%) were cervical (Supplementary Table 1). Seventeen patients had multiple episodes of gonococcal infection (Supplementary Table 2). Of these, most had 2 (8 patients [47%]) or 3 (6 patients [35%]) episodes and attended the clinic within 2 years (median, 231 days; range, 0–1071 days) of the previous diagnosis.

### Susceptibility to Antimicrobials

Disk diffusion testing [12] was used to determine susceptibility to penicillin and tetracycline, and the Etest [13] was used to determine minimal inhibitory concentrations (MICs) of ciprofloxacin, cefixime, penicillin, tetracycline, azithromycin, and doxycycline (bioMerieux, France). Gonococcal strains ATCC 31426 and ATCC 49226 [12] and WHO F, WHO G, WHO L, WHO O, and WHO P [13] were used as references.

### DNA Isolation and WGS

*N. gonorrhoeae* was grown overnight on Chocolate GC Selective Agar (Oxoid) in 5% CO_2_ at 37°C. Genomic DNA was extracted using the Wizard Genomic DNA Purification Kit (Promega). DNA was sequenced using Illumina HiSeq, and reads were assembled using the Velvet assembly program with VelvetOptimiser [14]. The resultant assemblies were uploaded to the pubMLST database (available at: http://www.pubmlst.org/neisseria), where data are publicly available, and linked to the European Nucleotide Archive (accession numbers ERR1143657–ERR976965; Supplementary Table 1).

### Whole-Genome Analysis

Whole-genome sequences were automatically annotated for defined loci, which identified alleles with ≥98% sequence identity. This enabled assignment of PorB and FetA types and multilocus sequence typing (MLST) sequence types (STs). The BIGsdb Genome comparator tool was used to compare WGS data, where 1668 loci were identified in the *N. gonorrhoeae* core genome (cgMLST *N. gonorrhoeae* v.1.0) [15]. Gonococci from the United States [10] and United Kingdom [11] and WHO reference strains [16] were compared to gonococci from coastal Kenya using the cgMLST scheme. Between 7 and 10 isolates from the most prevalent STs in the United States [10] and the United Kingdom [11] (ie, ST-1580, ST-1584, ST-1588, ST-1596, ST-1901, ST-7822, ST-8122, ST-9363, and ST-11990) were chosen in addition to strains from STs found also in coastal Kenya (ie, ST-1583, ST-1599, ST-1893, ST-1903, and ST-1931). Chromosomal and plasmid genes and intergenic regions implicated in gonococcal AMR are defined in the pubMLST *Neisseria* database [17]. The type of p*bla*TEM was determined by polymerase chain reaction as previously described [18]. Plasmid alignments were built using Easyfig [19].

## RESULTS

### Gonococcal Isolates in Coastal Kenya Are Distinct From Those in the Rest of the World

To characterize *N. gonorrhoeae* strains causing STI in coastal Kenya, we sequenced the genomes of 103 gonococcal isolates and analyzed them using the BIGSdb genomics platform hosted on PubMLST.org/neisseria. The resultant assemblies contained an average DNA length of 2211853 bp and 159 contigs (Supplementary Table 3). A total of 22 STs were found by MLST, with ST-1893 (n = 29), ST-1903 (n = 21), ST-1599 (n = 14), and ST-11366 (n = 11) the most prevalent (Supplementary Table 1).

**Figure 1.**
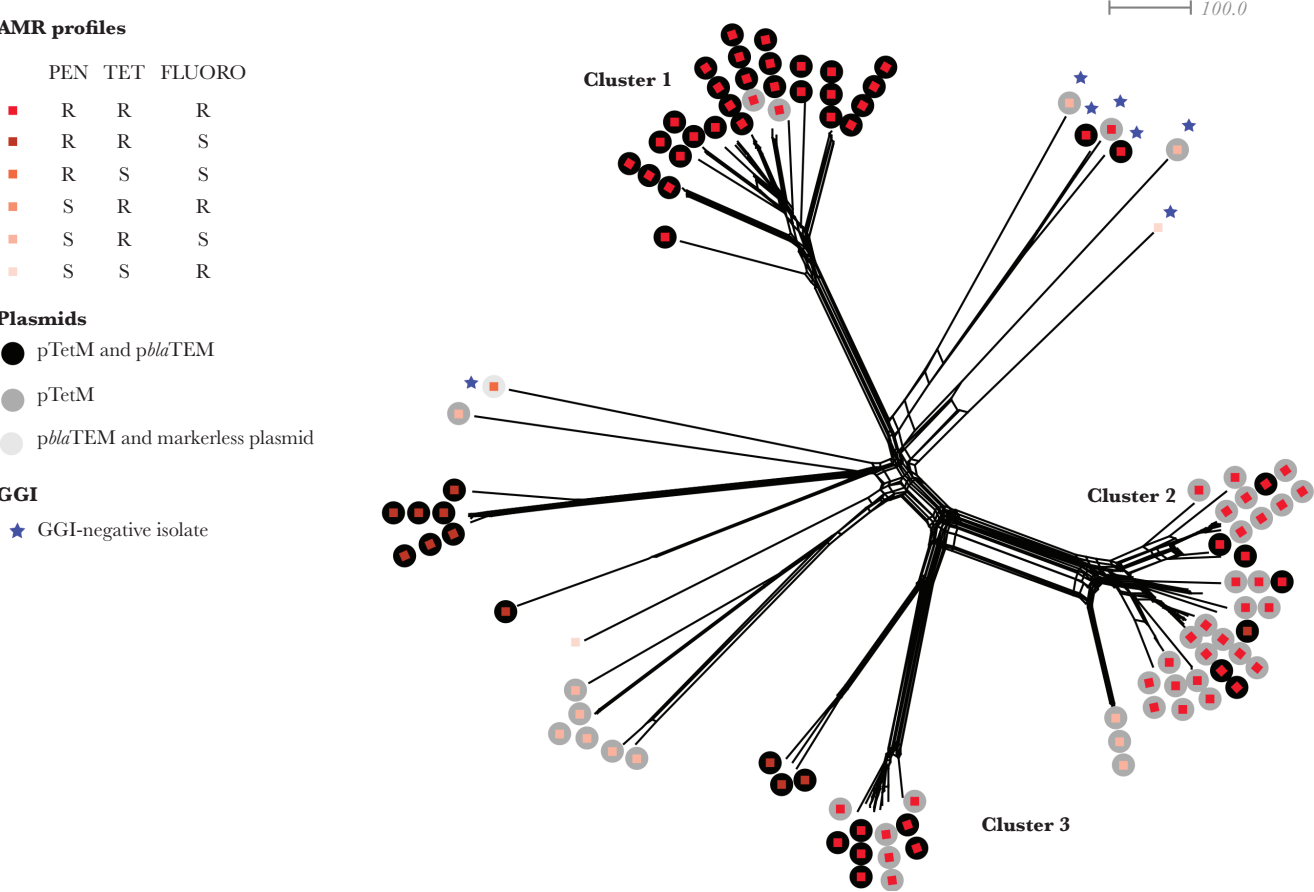
Whole-genome genealogy of *Neisseria gonorrhoeae* isolates in coastal Kenya. A Neighbor-Net graph depicting core genome multilocus sequence typing–based comparison of whole-genome sequencing data. Each branch represents one isolate, with squares and circles color-coded according to Antimicrobial resistance genotype and plasmid type, respectively. Clusters 1, 2, and 3 are indicated. Blue stars indicate the absence of gonococcal genetic island (GGI). FLUORO, fluoroquinolones; PEN, penicillin; R, resistant; S, susceptible; TET, tetracycline.

To understand phylogenetic relationships among isolates, we performed cgMLST analysis, which generated a star-burst phylogeny revealing the presence of 3 distinct clusters of isolates: cluster 1 (n = 30), including ST-1903; cluster 2 (n = 36), including ST-1893; and cluster 3 (n = 11), including ST-11366 (Figure 1). There was no association between HIV infection and cluster. The remaining 26 isolates did not belong to any cluster and were located on longer branches of the phylogenetic tree, consistent with them being distantly related and diverse.

**Figure 2.**
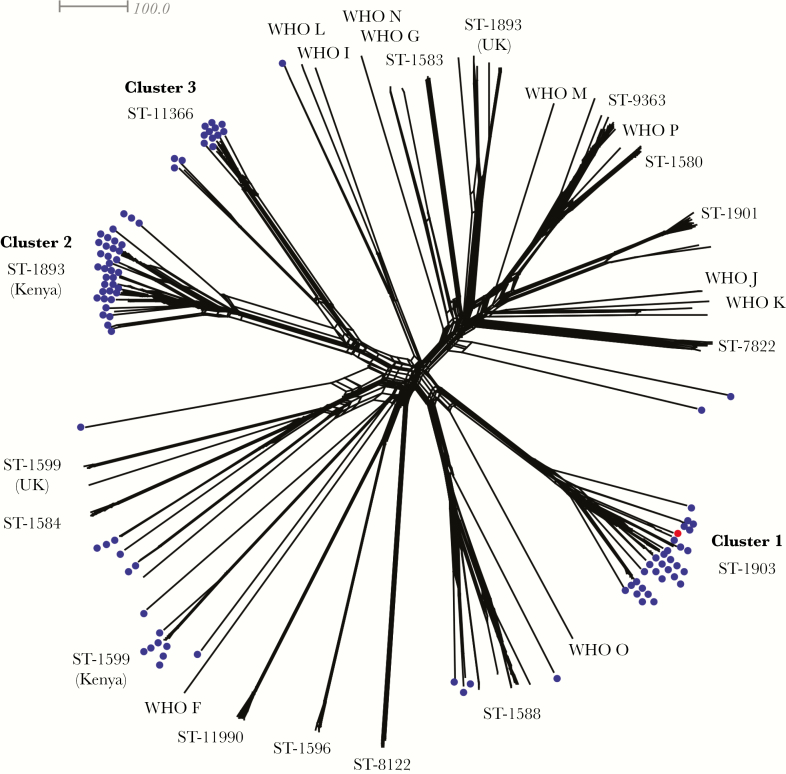
Whole-genome genealogy of *Neisseria gonorrhoeae* isolates in coastal Kenya and isolates from the United States [10] United Kingdom [11] and World Health Organization (WHO) reference strains [16]. A Neighbor-Net graph comparing WGS data on the basis of core genome multilocus sequence typing. Each branch represents 1 isolate, and their sequence types (STs) are indicated. The isolates from coastal Kenya are depicted with blue dots; the red dot represents a United Kingdom (UK) isolate belonging to ST-1903 [11].

To understand how gonococci from coastal Kenya compare with isolates from elsewhere in the world [10, 11], including WHO reference isolates [16], phylogenetic comparisons of gonococcal core loci were undertaken (Figure 2). Clusters 1, 2, and 3 were distinct from isolates commonly found in the United States and United Kingdom, such as ST-1901, ST-9363, and ST-11990 [10, 11]. Based on MLST, which compares fragments of 7 housekeeping genes, isolates belonging to ST-1599 and ST-1893, which are represented in our collection, are also found in the United Kingdom [11]. However, cgMLST provided greater resolution and demonstrated that the strains from the United Kingdom are distinct from those found in Kenya (Figure 2).

### High Prevalence of Mobile Genetic Elements Among Gonococcal Isolates in a High-Risk Group

**Figure 3.**
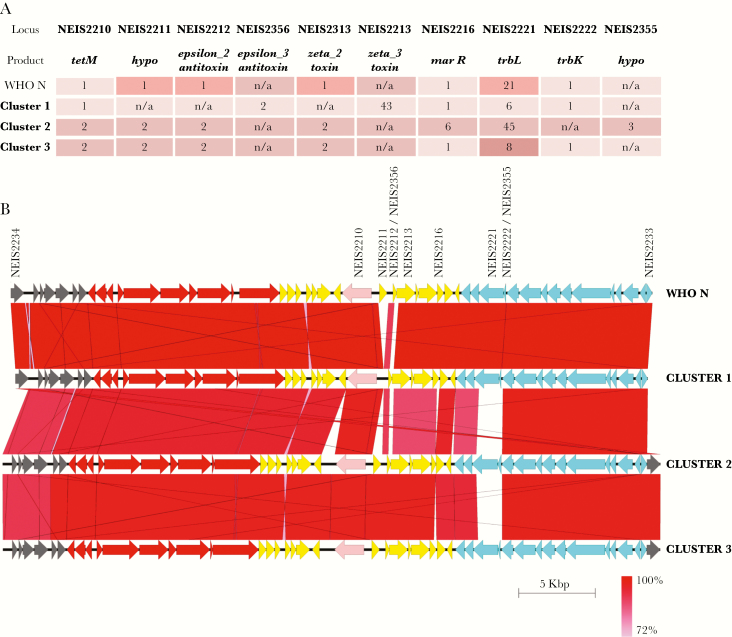
pTetM plasmids in coastal Kenya. *A*, Each cluster of isolates has a distinct allelic profile of loci involved in antimicrobial resistance (*tetM*), plasmid stability (ε/ζ toxin-antitoxin and *marR*), and mating pair formation (*trbK* and *trbL*). Only the most represented alleles in each cluster are listed. *B*, Alignment of mating pair formation (blue arrows), genetic load (yellow arrows), and conjugative transfer (red arrows) regions in pTetM from clusters 1, 2, and 3 and World Health Organization reference strain N (WHO N). The *tetM* allele is depicted in pink. Isolates used for alignment are 64500 (cluster 1), 43305 (cluster 2), and 64203 (cluster 3). Sequence similarity between genes is depicted as shades of red, as indicated at the bottom right corner of the figure. pTetM from WHO N, which carries a Dutch *tetM* allele, is used as a comparison in both panels. hypo, hypothetical protein; n/a, locus not present.

One hundred isolates (97%) harbored pTetM, a conjugative plasmid conferring tetracycline resistance. A further isolate, 45029 (Supplementary Table 1), contained the conjugative plasmid without *tetM*. Both types of *tetM* (NEIS2210), Dutch (allele 1) and American (allele 2) [20], were found: allele 1 was only found in strains belonging to cluster 1, while all other isolates contained allele 2. Detailed analysis of pTetM loci revealed that each cluster had a distinct allelic profile, regardless of the type of *tetM*. Major differences between plasmids were in loci involved in plasmid stability (ε/ζ toxin-antitoxin system and *marR* regulator) and mating pair formation (*trbK* and *trbL*; Figure 3) [20].

We examined patient records to see whether the strikingly high prevalence of pTetM in this population could be explained by antimicrobial use. Clinical data were available for 61 of 73 participants (83.6%) and indicated extensive use of doxycycline: 54 (88.5%) received doxycycline in the 6 months before diagnosis, while 50 (82%) received doxycycline on the day of diagnosis. We therefore examined whether pTetM confers resistance to doxycycline in a panel of representative strains (Supplementary Table 4). Of note, all pTetM-containing isolates were less susceptible to doxycycline (MIC range, 1.5–12 μg/mL) as compared to isolates without *tetM* (MIC range, from 0.19–0.38 μg/mL; Supplementary Table 4), confirming that pTetM confers doxycycline resistance. This is consistent with the high use of doxycycline selecting for the maintenance of pTetM.

Fifty-seven isolates (55%) contained the nonconjugative plasmid p*bla*TEM, all of which were of the African type (pJD5) [18]. The highest prevalence of p*bla*TEM was in isolates belonging to cluster 1 (28 of 30 [93%]; Table 1). In this cluster and in other distantly related isolates, allele 3 of *bla*TEM (NEIS2357), which encodes TEM-1 β-lactamase, was the most prevalent. We identified 2 novel *bla*TEM alleles, 10 and 12 (Table 1). These differed from allele 3 by a single amino acid insertion (Q5) and a single substitution (H6Y) for allele 10 or from allele 12 by a single substitution (A224T). Both these alleles were associated with high-level resistance to penicillin, with MICs ranging from 16 μg/mL (allele 12, strain 47547) to 192 μg/mL (allele 10, strains 42876 and 50659; Supplementary Table 4).

**Table 1.** Distribution of β-Lactamase Plasmids With Specific *bla*TEM Alleles

*bla*TEM Allele	Cluster 1, No. (%) (n = 30)^a^	Cluster 2, No. (%) (n = 36)	Cluster 3, No. (%) (n = 11)	Other, No. (%) (n = 26)^a^
**3**	28 (93)	1 (3)	1 (9)	10 (38)
**10**	0 (0)	6 (16)	5 (45)	3 (11)
**12**	0 (0)	0 (0)	0 (0)	1 (4)

^a^In 1 isolate in this group, the *bla*TEM allele could not be determined.

n = total number of isolates in this cluster.

We also detected a high prevalence of the gonococcal genetic island (GGI), a mobile genetic element encoding a type 4 secretion system implicated in DNA export [5]. The GGI was present in 97 isolates (94%; Figure 1). Compared with GGI-harboring strains, there are no polymorphisms in the *dif* insertion site and *xerC* and *xerD* site-specific recombinases [5] that could explain the absence of the GGI in the 6 strains lacking this mobile element.

### Chromosomally Encoded AMR Determinants in Kenyan Isolates

WGS analysis revealed that each cluster has a distinct genotypic AMR profile (Figure 1 and Table 2). All isolates contained chromosomal AMR determinants conferring resistance to fluoroquinolones and penicillin (Table 2). Nonsynonymous mutations in *gyrA* (NEIS1320), which confer resistance to fluoroquinolones (Table 3) [17], were highly prevalent in isolates from all 3 clusters: 28 of 30 (93%) in cluster 1, 28 of 36 (78%) in cluster 2, and 10 of 11 (91%) in cluster 3 (Table 2). Mutations in *parC* and *parE*, also associated with fluoroquinolone resistance [17], were not found.

**Table 2.** Chromosomal Antimicrobial Resistance Determinants Present in Each Cluster

Determinant	Cluster 1, No. (%) (n = 30)	Cluster 2, No. (%) (n = 36)	Cluster 3, No. (%) (n = 11)
**NEIS1320 (*gyrA***)	28 (93)	28 (78)	10 (91)
**NEIS1753 (*penA***)	30 (100)	30 (83)	0 (0)
**NEIS0414 (*ponA***)	30 (100)	35 (97)	11 (100)
**NEIS1635 (*mtrR***)	0 (0)	2 (5)	11 (100)
**pro_NEIS1635 (mtrR promoter region**)	18 (60)	0 (0)	0 (0)
**NEIS2020 (*porB***)	20 (66)	5 (14)	4 (36)

n = total number of isolates in this cluster.

**Table 3.** Antimicrobial Resistance (AMR) Alleles and Corresponding Mutations Conferring Resistance in *Neisseria gonorrhoeae* Isolates From This Study

Locus	AMR-Associated Amino Acid Substitution(s) in Kenyan Isolates	Allele(s) With Mutations Conferring AMR
**NEIS1320 (*gyrA***)	S91—F, D95—G/A	14, 234
**NEIS1753 (*penA***)	F504—L, P551—L	20, 23, 166, 228, 285, 294
**NEIS0414 (*ponA***)	L421—P	13
**NEIS1635 (*mtrR***)	Premature stop codons	423, 424, 427, 846, 847
**pro_NEIS1635 (mtrR promoter region**)	Adenine deletion in promoter region	3
**NEIS2020 (*porB***)	G120—D, A121—G/S/D	719, 826, 957, 1106, 1107, 1111, 1117, 1118, 1120, 1123, 1130, 1132, 1133, 1279, 1280, 1281, 1282, 1287, 1288
**NEIS2357 (*blaTEM***)	Not relevant	3, 10
**NEIS2210 (*tetM***)	Not relevant	1, 2

Isolates in clusters 1 and 2 had nonsynonymous substitutions in *penA* (NEIS1753) and *ponA* (NEIS0414; Tables 2 and 3), which confer resistance to β-lactams [17]. In addition, 20 of 30 (66%) and 18 of 30 (60%) in cluster 1 harbored AMR-associated mutations in *porB* (NEIS2020) and in the promoter of *mtrR* (*pro_NEIS1635*), respectively (Tables 2 and 3). No cluster 1 isolates and only 2 cluster 2 isolates harbored premature stop codons in *mtrR* (NEIS1635), which result in overexpression of the MtrCDE efflux pump (Tables 2 and 3) [17]. However, cluster 2 isolates had no AMR-associated mutations in the *mtrR* promoter (Table 2). In contrast, cluster 3 isolates did not harbor AMR-associated mutations in *penA*, but all contained AMR-associated mutations in *ponA* and harbored internal stop codons in *mtrR* (Table 2).

Different AMR characteristics were present in the remaining 26 more distantly related isolates (Figure 1). Sixteen of these isolates did not have any chromosomally mediated resistance (excluding resistance-associated mutations in *porB*) but possessed pTetM and p*bla*TEM (Figure 1).

We established AMR profiles by measuring MICs for tetracycline, azithromycin, and penicillin in 14 representative isolates. Phenotypic AMR profiles for the strains were concordant with our genotypic analysis (Supplementary Table 4).

Of note, all isolates in our study contained the *rpsJ* allele encoding S10 protein with Met^57^ mutation, which has been shown to increase resistance to tetracycline [21]. However, in our study, the MIC for tetracycline ranges from 0.25 μg/mL (for strains 65600 and 63179) to 16 μg/mL (for strains 64500, 42974, 64204, and 47547; Supplementary Table 4), suggesting that this mutation had no effect on the observed low- or high-level resistance to tetracycline.

No isolate harbored mutations in 23s ribosomal RNA and ribosomal protein S5 (NEIS0149), which are associated with resistance to azithromycin and spectinomycin, respectively [17]. Additionally, no isolates had mosaic *penA* alleles associated with resistance to third-generation cephalosporins [17]. Consistent with this, all 103 isolates were susceptible to cefixime (MIC range, 0.016–0.064 μg/mL), and a panel of representative isolates were susceptible to azithromycin (Supplementary Table 4).

### A Significant Proportion of Multiple Episodes of Gonorrhea are Caused by Closely Related Strains

**Figure 4.**
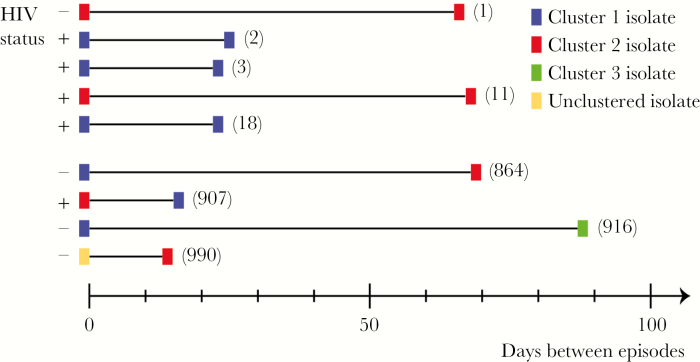
Gonococcal infections in patients with multiple episodes of gonorrhea occurring <100 days apart. Closely related isolates were obtained predominantly from human immunodeficiency virus (HIV)–positive individuals <100 days apart. In other infections, which occurred within a similar time frame, unrelated strains were recovered. Strains are marked with colored boxes, which refer to a cluster, as indicated in the legend. The distance between 2 strains is depicted in number of days between the 2 episodes of gonococcal disease in the same individual. The numbers in brackets refer to number of locus differences by cgMLST between the two strains. +, HIV positive at the time of strain isolation.

Seventeen patients had multiple episodes of gonorrhea (47 isolates; Supplementary Table 2). We performed cgMLST analysis to determine whether these episodes were caused by closely related or unrelated strains. Two isolates (64203 and 64204; Supplementary Table 2) were obtained from 1 individual on the same day from different sites (urethra and rectum) and exhibited only 1 difference by cgMLST, so they were likely the same strain. Most pairs of isolates differed by a median of 884 loci (range, 67–990 loci). However, 5 pairs of isolates (10 of 47 [21%]) from 4 patients had ≤18 locus differences by cgMLST (median, 3 loci; range, 1–18 loci; Supplementary Table 2), consistent with the multiple episodes resulting from inadequate treatment or reinfection from the same source. These closely related pairs of isolates were recovered from urethral swabs, except one (43346), which was from a rectal swab. Four of the closely related pairs of isolates were from 3 HIV-positive individuals, and the time between episodes ranged from 22 to 67 days (median, 24 days; Figure 4 and Supplementary Table 2).

## DISCUSSION

This study provides the first WGS analysis of gonococcal isolates from Africa where the highest rates of gonorrhea have been documented [7]. Comparison of genes core to the gonococcus revealed the presence of distinct gonococcal lineages clustering by AMR genotype, which are phylogenetically distinct from those found elsewhere in the world. Moreover, strains that are prevalent in other parts of the world and have spread globally [10, 11] were not found in coastal Kenya. Of note, the strains from our high-risk group possess a remarkably high frequency of mobile genetic elements.

Plasmid-mediated AMR among this collection is the highest observed in any gonococcal population, with 97% possessing pTetM and 55% possessing p*bla*TEM. The prevalence of pTetM varies between countries, ranging from 6% in cefixime-resistant isolates in the United States [10] to 73.3% in South Africa [22]. The unique lineages in coastal Kenya may have adaptive chromosomal mutations that compensate for the fitness costs that are often associated with harboring a plasmid [23]. The prevalence of pTetM is most likely a consequence of the high use of doxycycline in this cohort, as isolates with pTetM exhibit reduced susceptibility to this antibiotic. Where diagnostic facilities are limited, doxycycline is used in line with WHO guidelines for treatment of nongonococcal urethritis, proctitis, and cervicitis [24] and/or prior to laboratory confirmation of disease. pTetM is a conjugative plasmid [20], so it can rapidly disseminate in a population with appropriate selection pressure, such as the use of doxycycline. Furthermore, pTetM can facilitate the transfer of nonconjugative plasmids, such as p*bla*TEM, which are not self-mobilizable [6]. Therefore, use of doxycycline can coselect for p*bla*TEM and pTetM, resulting in spread of resistance to penicillin and tetracycline in a gonococcal population. Consistent with our findings, the frequency of p*bla*TEM mirrors that of pTetM in other gonococcal populations [10, 22, 25].

The prevalence of plasmids carrying *bla*TEM-1 is a significant concern because only a few single-nucleotide polymorphisms are required for TEM-1 to evolve into an extended-spectrum β-lactamase [26], which would result in rapidly transmissible cephalosporin resistance in gonococcus, a major public health threat [27]. Indeed, we found evidence of genetic alterations in TEM-1 with the occurrence of 2 novel *bla*TEM alleles (NEIS2357 alleles 10 and 12) associated with high-level resistance to penicillin. NEIS2357 allele 10 carries alterations in N-terminal signal peptide, which may enhance export of the enzyme into the periplasm, resulting in increased resistance [28]. A single substitution, A224T, is present in NEIS2357 allele 12; so far, this allele has only been observed in the laboratory and its effects on resistance are unknown [29].

Our results indicate that the use of doxycycline for empirical therapy of STIs might impact the nature and mechanisms of gonococcal AMR, and this should be considered when devising global treatment strategies for STIs. In addition, the high prevalence of p*bla*TEM in a high-risk population emphasizes the need for a combined therapy to prevent the emergence of mobile resistance to third-generation cephalosporins [30], even where local strains are susceptible to both cefixime and azithromycin, the WHO recommended therapies [31].

As well as plasmids, the GGI is highly prevalent in the gonococcal strains in this population. The GGI is associated with the spread of AMR through unknown mechanisms and is thought to have contributed to the expansion of the ST-1901 lineage in the western hemisphere [17]. Interestingly, 94% of isolates in our study harbored the GGI and plasmids, unlike other gonococcal populations, where they tend to be mutually exclusive [17].

In this study, we characterized gonococcal isolates from patients with multiple episodes of gonorrhea. Four patients (23% of those with repeated episodes), 3 of whom were HIV positive, were infected with closely related strains at different times. This frequency differs from a larger study in the United Kingdom, in which only 5% of multiple episodes were caused by closely related strains [11]. The reisolation of phylogenetically similar strains from an individual, in one instance up to 67 days later, indicates that the strain is either undergoing intense transmission or that the patient has not been treated adequately. These findings highlight the need for appropriate test of cure and intense contact tracing to ensure that initial infections are eliminated in high-risk populations.

Core groups of high-risk individuals have the potential to transmit gonorrhea and other STIs to a large number of sex partners [9]. Comprehensive monitoring, treatment, and understanding of gonococcal transmission in high-risk groups are therefore essential for the effective management and control of gonorrhea in such settings. Africa is an area with high prevalence of gonococcal disease [7], and it is therefore critical to continue the surveillance of AMR on this continent and monitor the emergence of novel gonococcal lineages. Data presented here provide compelling evidence that gonococcal populations become structured by antibiotic use as a result of selection pressure, and they emphasize the need for a global strategy to effectively control this infection [27]. Our data highlight the need to acquire globally distinct gonococcal collections, in which antimicrobial selection pressures will be different, thus enhancing our understanding of the emergence of AMR in this important pathogen.

## Supplementary Data

Supplementary materials are available at *The Journal of Infectious Diseases* online. Consisting of data provided by the authors to benefit the reader, the posted materials are not copyedited and are the sole responsibility of the authors, so questions or comments should be addressed to the corresponding author.

Supplementary Table 1

Supplementary Table 2

Supplementary Table 3

Supplementary Table 4

## Notes

***Acknowledgments.***  We thank Prof Magnus Unemo from the WHO Collaborating Centre for Gonorrhoea and Other STIs (Örebro, Sweden), for the *N. gonorrhoeae* WHO reference strains.

This report was published with permission from the KEMRI.

***Financial support.*** This work was supported by Oxford Martin School and the Wellcome Trust (grants 100298/Z/13/Z and 203077/Z/16/Z).

***Potential conflicts of interest.*** All authors: No reported conflicts of interest. All authors have submitted the ICMJE Form for Disclosure of Potential Conflicts of Interest. Conflicts that the editors consider relevant to the content of the manuscript have been disclosed.

Presented in part: 7th Congress of European Microbiologists, Valenica, Spain, 9–13 July 2017 (abstract FEMS7-3112); 14th Annual Meeting of the European Institute of Microbiology and Infectious Diseases, Oxford, United Kingdom, 27–29 September 2017 (abstract W10).

## References

[1] World Health Organization. Global action plan to control the spread and impact of antimicrobial resistance in *Neisseria gonorrhoeae* http://www.who.int/reproductivehealth/publications/rtis/9789241503501/en/. Accessed 8 May 2018.

[2] Unemo M, Del Rio C, Shafer WM Antimicrobial resistance expressed by *Neisseria gonorrhoeae*: a major global public health problem in the 21st century. Microbiol Spectr 2016; 4:doi: 10.1128/microbiolspec.EI10-0009-2015. 10.1128/microbiolspec.EI10-0009-2015 PMC4920088 27337478

[3] Galvin SR, Cohen MS The role of sexually transmitted diseases in HIV transmission. Nat Rev Microbiol 2004; 2:33–42. 15035007 10.1038/nrmicro794

[4] World Health Organization. Global priority list of antibiotic-resistant bacteria to guide research, discovery, and development of new antibiotics http://www.who.int/medicines/publications/global-priority-list-antibiotic-resistant-bacteria/en/. Accessed 8 May 2018.

[5] Cehovin A, Lewis SB Mobile genetic elements in *Neisseria gonorrhoeae*: movement for change. Pathog Dis 2017; 75:doi: 10.1093/femspd/ftx071. 10.1093/femspd/ftx071 28645177

[6] Roberts M, Falkow S In vivo conjugal transfer of R plasmids in *Neisseria gonorrhoeae*. Infect Immun 1979; 24:982–4. 112063 10.1128/iai.24.3.982-984.1979 PMC414408

[7] World Health Organization. Global incidence and prevalence of selected curable sexually transmitted infections—2008 http://www.who.int/reproductivehealth/publications/ rtis/stisestimates/en/. Accessed 8 May 2018.

[8] Sanders EJ, Thiong’o AN, Okuku HS, et al High prevalence of Chlamydia trachomatis and *Neisseria gonorrhoeae* infections among HIV-1 negative men who have sex with men in coastal Kenya. Sex Transm Infect 2010; 86:440–1. 20656722 10.1136/sti.2010.043224

[9] Lewis DA The role of core groups in the emergence and dissemination of antimicrobial-resistant N gonorrhoeae. Sex Transm Infect 2013; 89(Suppl 4):iv47–51. 24243880 10.1136/sextrans-2013-051020

[10] Grad YH, Kirkcaldy RD, Trees D, et al Genomic epidemiology of *Neisseria gonorrhoeae* with reduced susceptibility to cefixime in the USA: a retrospective observational study. Lancet Infect Dis 2014; 14:220–6. 24462211 10.1016/S1473-3099(13)70693-5 PMC4030102

[11] De Silva D, Peters J, Cole K, et al Whole-genome sequencing to determine transmission of *Neisseria gonorrhoeae*: an observational study. Lancet Infect Dis 2016; 16:1295–303. 27427203 10.1016/S1473-3099(16)30157-8 PMC5086424

[12] Centers for Disease Control and Prevention. Disk diffusion susceptibility testing https://www.cdc.gov/std/gonorrhea/lab/diskdiff.htm. Accessed 8 May 2018.

[13] World Health Organization. Laboratory diagnosis of sexually transmitted infections, including human immunodeficiency virus http://who.int/reproductivehealth/publications/rtis/ 9789241505840/en/. Accessed 8 May 2018.

[14] Bratcher HB, Corton C, Jolley KA, Parkhill J, Maiden MC A gene-by-gene population genomics platform: de novo assembly, annotation and genealogical analysis of 108 representative *Neisseria meningitidis* genomes. BMC Genomics 2014; 15:1138. 25523208 10.1186/1471-2164-15-1138 PMC4377854

[15] Harrison OB, Cole K, Peters J, et al Genomic analysis of urogenital and rectal *Neisseria meningitidis* isolates reveals encapsulated hyperinvasive meningococci and coincident multidrug-resistant gonococci. Sex Transm Infect 2017; 93:445–51. 10.1136/sextrans-2016-052781 PMC5574384 28137933

[16] Unemo M, Golparian D, Sánchez-Busó L, et al The novel 2016 WHO *Neisseria gonorrhoeae* reference strains for global quality assurance of laboratory investigations: phenotypic, genetic and reference genome characterization. J Antimicrob Chemother 2016; 71:3096–108. 27432602 10.1093/jac/dkw288 PMC5079299

[17] Harrison OB, Clemence M, Dillard JP, et al Genomic analyses of *Neisseria gonorrhoeae* reveal an association of the gonococcal genetic island with antimicrobial resistance. J Infect 2016; 73:578–87. 27575582 10.1016/j.jinf.2016.08.010 PMC5127880

[18] Dillon JR, Li H, Yeung K, Aman TA A PCR assay for discriminating *Neisseria gonorrhoeae*beta-lactamase-producing plasmids. Mol Cell Probes 1999; 13:89–92. 10208798 10.1006/mcpr.1998.0216

[19] Sullivan MJ, Petty NK, Beatson SA Easyfig: a genome comparison visualizer. Bioinformatics 2011; 27:1009–10. 21278367 10.1093/bioinformatics/btr039 PMC3065679

[20] Pachulec E, van der Does C Conjugative plasmids of *Neisseria gonorrhoeae*. PLoS One 2010; 5:e9962. 20376355 10.1371/journal.pone.0009962 PMC2848598

[21] Hu M, Nandi S, Davies C, Nicholas RA High-level chromosomally mediated tetracycline resistance in *Neisseria gonorrhoeae* results from a point mutation in the rpsJ gene encoding ribosomal protein S10 in combination with the mtrR and penB resistance determinants. Antimicrob Agents Chemother 2005; 49:4327–34. 16189114 10.1128/AAC.49.10.4327-4334.2005 PMC1251527

[22] Fayemiwo SA, Müller EE, Gumede L, Lewis DA Plasmid-mediated penicillin and tetracycline resistance among *Neisseria gonorrhoeae* isolates in South Africa: prevalence, detection and typing using a novel molecular assay. Sex Transm Dis 2011; 38:329–33. 21042234 10.1097/OLQ.0b013e3181fc695a

[23] Sommer MOA, Munck C, Toft-Kehler RV, Andersson DI Prediction of antibiotic resistance: time for a new preclinical paradigm?Nat Rev Microbiol 2017; 15:689–96. 28757648 10.1038/nrmicro.2017.75

[24] World Health Organization. WHO guidelines for the treatment of *Chlamydia trachomatis* 2016 http://www.who.int/reproductivehealth/publications/rtis/chlamydia-treatment-guidelines/en/. Accessed 8 May 2018. 27559553

[25] Zheng H, Wu X, Huang J, et al The prevalence and epidemiology of plasmid-mediated penicillin and tetracycline resistance among *Neisseria gonorrhoeae* isolates in Guangzhou, China, 2002-2012. BMC Infect Dis 2015; 15:412. 26453557 10.1186/s12879-015-1148-9 PMC4600260

[26] Arlet G, Goussard S, Courvalin P, Philippon A Sequences of the genes for the TEM-20, TEM-21, TEM-22, and TEM-29 extended-spectrum beta-lactamases. Antimicrob Agents Chemother 1999; 43:969–71. 10103213 10.1128/aac.43.4.969 PMC89239

[27] Wi T, Lahra MM, Ndowa F, et al Antimicrobial resistance in *Neisseria gonorrhoeae*: global surveillance and a call for international collaborative action. PLoS Med 2017; 14:e1002344. 28686231 10.1371/journal.pmed.1002344 PMC5501266

[28] Goldsmith M, Tawfik DS Potential role of phenotypic mutations in the evolution of protein expression and stability. Proc Natl Acad Sci U S A 2009; 106:6197–202. 19339491 10.1073/pnas.0809506106 PMC2669386

[29] Kather I, Jakob RP, Dobbek H, Schmid FX Increased folding stability of TEM-1 beta-lactamase by in vitro selection. J Mol Biol 2008; 383:238–51. 18706424 10.1016/j.jmb.2008.07.082

[30] Unemo M Current and future antimicrobial treatment of gonorrhea—the rapidly evolving *Neisseria gonorrhoeae* continues to challenge. BMC Infect Dis 2015; 15:364. 26293005 10.1186/s12879-015-1029-2 PMC4546108

[31] World Health Organization. WHO guidelines for the treatment of *Neisseria gonorrhoeae* 2016 http://www.who.int/reproductivehealth/publications/rtis/gonorrhoea-treatment-guidelines/en/. Accessed 8 May 2018. 27512795

